# Diagnostic accuracy of postmortem computed tomography for bleeding source determination in cases with hemoperitoneum

**DOI:** 10.1007/s00414-020-02472-0

**Published:** 2021-01-07

**Authors:** Vasiliki Chatzaraki, Michael J. Thali, Garyfalia Ampanozi

**Affiliations:** 1 Department of Forensic Medicine and Imaging, Institute of Forensic Medicine, University of Zurich, Winterthurerstrasse 190/52, CH-8057 Zurich, Switzerland; 2 Department of Radiology, Kantonsspital Baden AG, Im Ergel 1, CH-5404 Baden, Switzerland

**Keywords:** Hemoperitoneum, Abdominal hemorrhage, Bleeding source, Forensic radiology, Autopsy, Postmortem computed tomography (PMCT)

## Abstract

**Aim:**

The aim of this retrospective study was to determine the accuracy of postmortem computed tomography and different radiological signs for the determination of the bleeding source in cases with hemoperitoneum confirmed at autopsy.

**Methods:**

Postmortem computed tomography data of consecutive cases with hemoperitoneum confirmed at autopsy were reviewed by two raters, blinded to the autopsy findings. The determination of possible bleeding sources was based on the presence of the sentinel clot sign, blood or sedimented blood surrounding an organ, intraparenchymal abnormal gas distribution, and parenchymal disruption. The bleeding source and the cause of hemoperitoneum (traumatic, surgical, natural, or resuscitation) as reported in the autopsy report were noted. The survival intervals of the deceased were calculated when information about the time of an incident related to death was available in the autopsy reports.

**Results:**

Eighty-five cases were included in the study. Postmortem computed tomography showed 79% sensitivity and 92.1% specificity for the detection of the bleeding source. The sentinel clot sign was associated with surgical or natural causes of hemoperitoneum and longer survival intervals. Sedimented blood around the bleeding source was associated with resuscitation. Abnormal gas distribution within organs and combination of multiple radiological signs provided higher sensitivity.

**Conclusion:**

Postmortem computed tomography provides moderate sensitivity and high specificity for determining the bleeding source in cases with hemoperitoneum. Different PMCT signs are associated with different causes of hemoperitoneum and survival intervals.

## Introduction

Hemoperitoneum can be traumatic, spontaneous, or iatrogenic. Traumatic hemoperitoneum can be the result of solid organ, vasculature, bowel, mesenteric, or bladder injuries with the spleen and liver being the most frequently injured organs by blunt force and penetrating trauma [1–3]. Spontaneous hemoperitoneum has visceral organ-, gynecologic-, vascular-, and coagulopathy-related causes [4]. Iatrogenic causes include any surgical procedure performed in the intraperitoneal cavity usually combined with postoperative anticoagulation therapy [4].

In the clinical setting, non-contrasted computed tomography (CT) comprises an important screening tool for trauma management [5] and provides high sensitivity for the detection of small intraperitoneal effusions [1]. CT also provides high specificity and negative predictive value for the decision-making process regarding whether a liver injury requires intervention [6]. Radiological findings related to hemoperitoneum are intraperitoneal blood accumulation [1], the sentinel clot sign (high-attenuation blood clot adherent to an injured organ) [1, 7, 8], and directly visible organ injuries (parenchymal inhomogeneities, contusions, lacerations, hematomas) [1].

Blood usually has higher density than other intraperitoneal fluids (around 30–45 Hounsfield units (HU)) [1]. The sentinel clot sign is an indirect clue for determining the bleeding source in cases of hemoperitoneum [1, 4, 9, 10]. It is characterized by a focal heterogeneous area of clotted blood, which is denser than the rest of the fluid in the abdomen, indicating the site of the organ or vessel injured [7, 8]. The mean density of clotted blood is approximately within the range of 45–70 HU.

In the forensic routine, unenhanced postmortem CT (PMCT) is increasingly used to supplement autopsy. Postmortem computed tomography angiography (PMCTA) provides high sensitivity for the detection of essential findings regarding vessels and organ lesions; however, it is more time-consuming and therefore is mainly applied as a targeted examination in the forensic routine [11, 12]. Compared to antemortem CT, PMCTA, and autopsy, PMCT shows a more limited efficacy for diagnosing or ruling out abdominal injuries and particularly small ones [13, 14]. However, the sensitivity of PMCT for determination of hemoperitoneum is high [15], and the more life-threatening an abdominal injury, the higher the sensitivity [16]. Typical radiological findings of hemoperitoneum can be observed also postmortem, like blood surrounding an injured organ [15], sentinel clot [17, 18], parenchymal inhomogeneity, and abnormal gas distribution not following the vascular tree within solid organs [15]. Blood appears different on PMCT compared to antemortem imaging because of the postmortem changes and blood sedimentation [19, 20].

The primary aim of this study was to determine the diagnostic accuracy of PMCT and specific radiological signs for the determination of the intra-abdominal bleeding source in cases with hemoperitoneum confirmed at autopsy. Secondary aim was to examine PMCT accuracy for the detection of the bleeding source with regard to raters’ confidence for the radiological diagnosis, the cause of bleeding, and the survival interval.

## Materials and methods

### Subjects

All consecutive cases with age ≥ 18 years and postmortem interval ≤ 4 days based on the estimation of time of death [21–24] that underwent pre-autopsy PMCT and full forensic autopsy from January 1, 2011, to December 31, 2018, were retrospectively reviewed in our database. Autopsy reports were reviewed for recorded presence of hemoperitoneum. In cases with reported hemoperitoneum, the reports were further assessed for the bleeding source. Only cases with hemoperitoneum and the bleeding source at autopsy clearly found to be at least one of the following organs were included in the study: liver, spleen, right kidney, left kidney, pancreas, right adrenal gland, left adrenal gland, any abdominal artery. Cases with blood exclusively in the retroperitoneal space and not in the peritoneal cavity at autopsy were not included in this study. Organ findings at autopsy not considered as possible bleeding source, for example subcapsular hematomas of the liver, were not considered as relevant for this study. Exclusion criteria contained the following: (1) cases with other than pure blood accumulation in the abdomen, (2) not clearly stated bleeding source in the autopsy report, (3) cases with bowel and/or mesenterial rupture as bleeding site, (4) cases with intraperitoneal foreign bodies (including knives, projectiles, medical gauzes), (5) cases with gunshot, sharp force and fire injuries of the abdomen, and (6) destructing trauma with open injury of the peritoneum and/or abdominal organs’ dislocations.

During the autopsy reports’ review, the organs comprising bleeding sources were noted for every case. Additionally, the cause of bleeding was noted for every bleeding source as among traumatic, surgical/natural, or caused by resuscitation efforts. For the cases with traumatic and surgical/natural causes, the survival interval between the related incident and death was calculated, when applicable, based on the information given. The cases were divided into two categories of survival interval: ≤ 1 h and > 1 h.

### Imaging protocol – PMCT review

PMCT was performed on a 128-slice scanner (SOMATOM Definition Flash, Siemens Healthineers, Erlangen, Germany), with the bodies in supine position, using automatic dose modulation (CARE Dose 4D™, Siemens Healthineers, Erlangen, Germany). Imaging parameters were as follows [25]: tube voltage 120 kV, slice collimation 128 × 0.6 mm. Image reconstructions of the abdomen were performed [25], with a slice thickness of 1.0 mm and an increment of 0.6 mm. The arms were elevated if possible, in order to avoid artifacts.

Two raters (a forensic pathology trainee with 2.5 years’ experience and a board-certified forensic pathologist with 11 years’ experience in forensic radiology) evaluated the PMCT data in consensus reading for the radiological determination of the bleeding source based on the presence of the following five radiological signs: (1) sentinel clot sign (high-attenuation irregular fluid accumulation/blood clot around an organ or vessel while the rest fluid in the abdomen provides lower attenuation values); (2) hyperdense fluid (blood) around the organ (homogeneous fluid accumulation without internal density deviations); (3) sedimented blood around the organ (fluid-fluid level); (4) abnormal gas distribution within solid organs not following the vascular tree; and (5) parenchymal disruptions/lacerations. The raters were blinded to the autopsy findings during PMCT review. After the radiological determination of the possible bleeding source, the raters rated the confidence of every diagnosis based on a 3-point confidence-level Likert scale (level 1 = the organ could be bleeding source, level 2 = the organ is probably bleeding source, level 3 = the organ is definitely a bleeding source).

### Bleeding source determination based on PMCT signs

The sensitivity (SEN), specificity (SPC), positive predictive (PPV), negative predictive value (NPV), and positive and negative likelihood ratios of PMCT for the detection of the bleeding source were calculated independently of organ, as well as organ-dependently. Autopsy was considered the reference test.

The proportions of the true positive (TP) and false positive (FP) signs were calculated with respect to the distinct PMCT signs.

For the TP bleeding sources on PMCT, the relationships between survival interval and PMCT sign and between cause of the bleeding and PMCT sign were investigated.

### Raters’ confidence for detection of bleeding sources on PMCT

The confidence levels given by the raters were assessed with regard to the distinct PMCT signs. The relationship of the three confidence levels with the TP or FP diagnosis was investigated independently of the different radiological signs.

### Radiological signs with regard to cause of intra-abdominal bleeding

The distribution of the TP bleeding sources on PMCT in the three different cause of bleeding groups (trauma/surgical-natural/resuscitation) was examined. The frequencies of the radiological signs were compared among the different cause of bleeding groups.

### Survival interval with regard to performance of PMCT and radiological signs

For the cases with traumatic and surgical/natural causes of bleeding and available information in the autopsy reports, the survival interval was calculated and groups of ≤ 1 h and > 1 h were created. For the positive organs on PMCT, by which bleeding was caused by the abovementioned causes (and not by resuscitation), the proportions of TP and FP findings were compared between the two survival interval groups.

For the TP findings, the proportions of the different radiological signs were compared between the two different survival interval groups.

### Statistical analysis

Statistical analysis was performed with the SPSS Statistical Package (© IBM, SPSS 20, Chicago, IL, USA). Chi-square tests and Fischer’s exact tests of independence were used to compare proportions of nominal variables between nominal variables’ groups. *P* values less than 0.05 indicated statistical significance. Excel (Microsoft Excel, © 2010 Microsoft Corporation, USA) was used for creating graphs.

## Results

### Subjects

One hundred thirty-six cases with hemoperitoneum at autopsy were identified in the database. Fifty-one were excluded; thus, 85 cases were included in the study. Sixty-three (74%) concerned males (age 49 ± 19 years) and 22 (26%) females (age 59 ± 16 years). From the 85 cadavers included in the study, 57 (67%) were scanned with the arms elevated during PMCT; in the rest 28 (33%), elevating the arms was avoided because of extended external injuries or postmortem rigidity.

From the 679 assessed organs (85 cases × 8 organs/case = 680 − 1 case of absent left kidney = 679), 124 were determined as bleeding sources at autopsy (livers 60, spleens 40, right kidneys 4, left kidneys 6, pancreas 0, right adrenal glands 2, left adrenal glands 1, vessels 11: abdominal aorta 7, splenic artery 1, renal artery 1, external iliac artery 1, femoral artery 1). For 104 (84%) of the 124 bleeding sites, the cause was traumatic, for 12 (10%) surgical or natural, and the rest 8 (6%) were caused by resuscitation measures. Calculation of the survival interval of the subjects was applicable for 92 out of the 124 bleeding sites. Seventy-nine bleeding sites were associated with survival interval ≤ 1 h and 13 to > 1 h.

### Bleeding source determination based on PMCT signs

The overall performance of PMCT for the detection of the bleeding source found at autopsy organ-dependently, as well as independently of organ, is presented in Table 1. From the 124 bleeding sites, 26 were missed by PMCT (false negatives). Overall SEN of PMCT for the detection of an abdominal bleeding source was 79% (95% CI 70.8 to 85.8) (Table 2). From the 555 organs without pathological findings at autopsy, PMCT was positive for 44 (FP) (Table 1). Overall SPC was 92.1% (95% CI 89.5 to 94.2) (Table 2).

**Table 1:** Organ-based and organ-independent (total) 2 × 2 table evaluating the number of the organs determined as positive or negative bleeding source on PMCT with regard to autopsy results

		Autopsy +	Autopsy −	Total
Liver	PMCT +	47	3	50
	PMCT −	13	22	35
	Total	60	25	85
Spleen	PMCT +	31	22	53
	PMCT −	9	23	32
	Total	40	45	85
Kidney right	PMCT +	4	10	14
	PMCT −	0	71	71
	Total	4	81	85
Kidney left	PMCT +	5	4	9
	PMCT −	1	74	75
	Total	6	78	84
Pancreas	PMCT +	0	4	4
	PMCT −	0	81	81
	Total	0	85	85
Adrenal gland right	PMCT +	1	0	1
	PMCT −	1	83	84
	Total	2	83	85
Adrenal gland left	PMCT +	0	0	0
	PMCT −	1	84	85
	Total	1	84	85
Vessel	PMCT +	10	1	11
	PMCT −	1	73	74
	Total	11	74	85
Total	PMCT +	98	44	142
	PMCT −	26	511	537
	Total	124	555	679

*PMCT +*, organs with at least one radiological sign on PMCT; *PMCT −*, organs without radiological signs on PMCT; *AUT +*, organs positive at autopsy; *AUT −*, organs intact at autopsy

In each organ group, 85 organs were evaluated (one organ/case) except of the left kidney where in one case it was absent

**Table 2:** Sensitivity (*SEN*), specificity (*SPC*), positive and negative predictive value (*PVV* and *NPV*), and positive (+) and negative (−) likelihood ratios of PMCT for the detection of the bleeding source in cases with hemoperitoneum independently of organ (row at the bottom-total) and organ-dependently

	SEN (%) (95% CI)	SPC (%) (95% CI)	PPV (%) (95% CI)	NPV (%) (95% CI)	+ Likelihood ratio (95% CI)	− Likelihood ratio (95% CI)
Liver	78.3 (65.8, 87.9)	88 (68.8, 97.5)	94 (84.3, 97.9)	62.9 (50.6, 73.7)	6.53 (2.24, 19.03)	0.25 (0.15, 0.41)
Spleen	77.5 (61.6, 89.2)	51.1 (35.8, 66.3)	58.5 (50, 66.5)	71.9 (57.4, 82.9)	1.59 (1.13, 2.23)	0.44 (0.23, 0.84)
Kidney right	100 (39.8, 100)	87.7 (78.5, 93.9)	28.6 (18.3, 41.7)	100 (−)	8.1 (4.5, 14.5)	0
Kidney left	83.3 (35.9, 99.6)	94.9 (87.5, 98.6)	55.6 (31.1, 77.6)	98.7 (92.5, 99.8)	16.3 (5.9, 45)	0.18 (0.03, 1.05)
Kidney (right + left)	90 (55.5, 99.75)	91.2 (85.7, 95.1)	39.1 (27.2, 52.5)	99.3 (95.8, 99.9)	10.2 (5.9, 17.6)	0.11 (0.02, 0.7)
Pancreas	–	95.3 (88.4, 98.7)	–	–	–	1.05
Adrenal gland right	50 (1.3, 98.7)	100 (95.7, 100)	100	98.8 (95.4, 99.7)	–	0.5 (0.13, 2)
Adrenal gland left	0 (0, 97.5)	100 (95.7, 100)	–	98.8 (93.6, 99.9)	–	1 (1, 1)
Adrenal gland (right + left)	33.3 (0.8, 90.6)	100 (97.8, 100)	100	98.8 (97.4, 99.5)	–	0.67 (0.3, 1.5)
Vessel	90.9 (58.7, 99.8)	98.7 (92.7, 99.8)	90.9 (58.6, 98.6)	98.7 (91.9, 99.8)	67.3 (9.5, 475.5)	0.09 (0.01, 0.6)
Total	79 (70.8, 85.8)	92.1 (89.5, 94.2)	69 (62.3, 75)	95.2 (93.3, 96.5)	10 (7.4, 13.5)	0.23 (0.16, 0.32)

From the 142 positives on PMCT (98 TP + 44 FP), 39 were considered as positive because of present sentinel clot sign, 54 because of blood collection surrounding the related organ, 13 because of sedimented blood around the organ, 23 because of intraparenchymal abnormal gas distribution, and 11 based on combinations of signs (Table 3).

**Table 3:** The distribution of the organs with positive radiological sign/signs on PMCT in the true positive (TP = PMCT+, AUT+) and false positive (FP = PMCT+, AUT−) groups with regard to the different radiological signs

	TP	FP	Total
Sentinel clot (*n* organs - %)	26 (66.7%)	13 (33.3%)	39 (100%)
Blood (*n* organs - %)	33 (61.1)	21 (38.9)	54 (100%)
Sedimented blood (N organs - %)	7 (53.8%)	6 (46.2%)	13 (100%)
Gas distribution (*n* organs - %)	21 (91.3%)	2 (8.7%)	23 (100%)
Parenchymal disruption (*n* organs - %)	1 (50%)	1 (50%)	2 (100%)
Combination (*n* organs - %)	11 (100%)	0	11 (100%)
Total (*n* organs - %)	99 (69.7%)	43 (30.3%)	142 (100%)

#### Sentinel clot sign

**Fig. 1:**
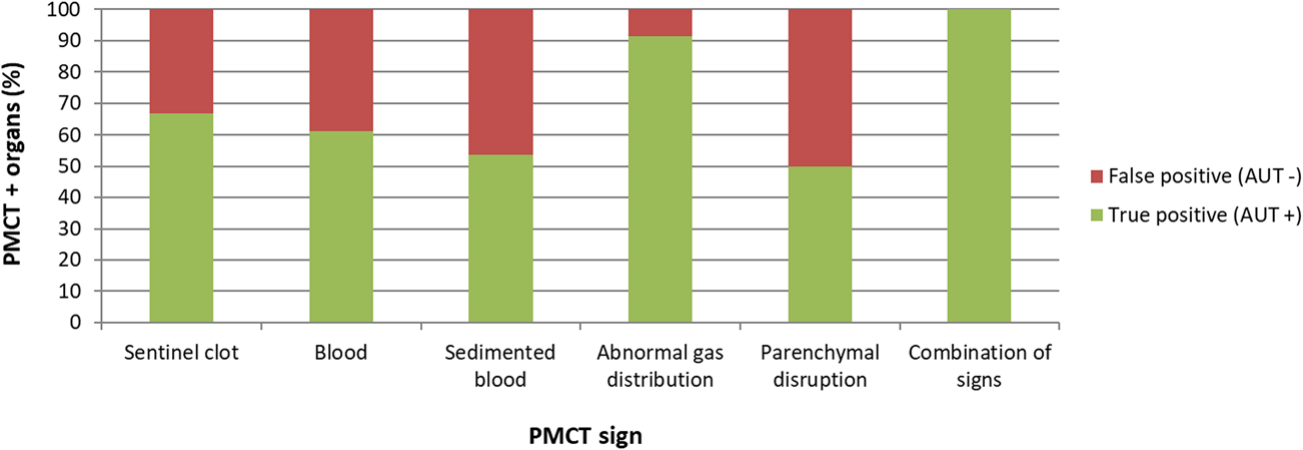
Bar chart depicting the frequencies (%) of the organs determined as positive on PMCT, i.e., the organs with at least one radiological sign on PMCT (“PMCT + organs”; true positive with green and false positive with red) with regard to the different radiological signs

**Fig. 2:**
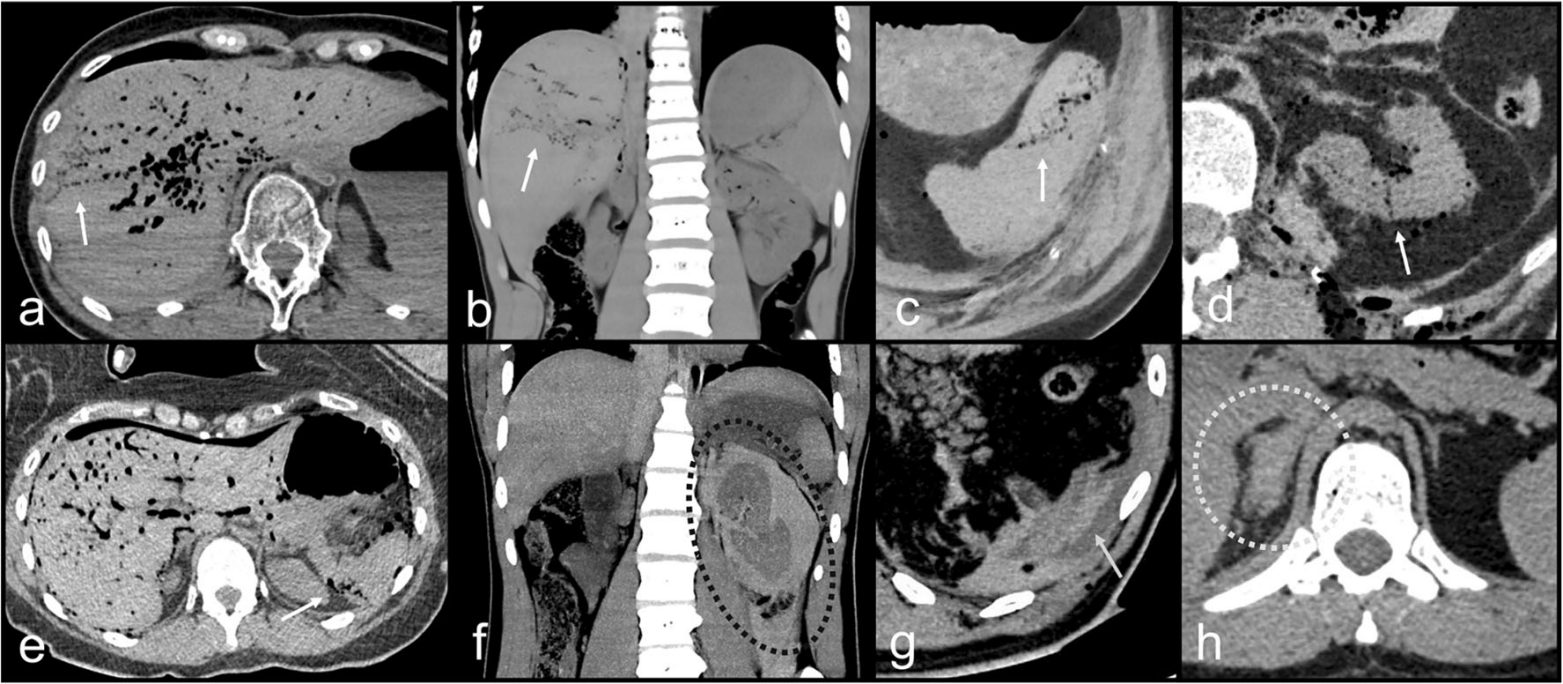
PMCT multiplanar reconstructions (MPR) in different cases with true positive abnormal findings. **a** PMCT axial slice through the abdomen; true positive abnormal gas distribution not following the vascular tree within the liver. **b** PMCT coronal slice through the abdomen; true positive abnormal gas distribution within the liver not following the vascular tree. PMCT axial slices through the spleen (**c**) and left kidney (**d**); true positive abnormal gas distributions within a spleen and a kidney. **e** PMCT axial slice through the abdomen; true positive abnormal gas distribution within the spleen. Note the prominent but normally distributed gas in the liver within the vascular tree. Liver was true negative in this case. **f** PMCT coronal slice through the abdomen; extended parenchymal disruption of the left kidney with sentinel clot sign. **g** PMCT axial slice through the spleen; sentinel clot sign. **h** PMCT axial slice through the right adrenal gland; blood around the right adrenal gland (note the difference with the left one) as true positive sign on PMCT

**Fig. 3:**
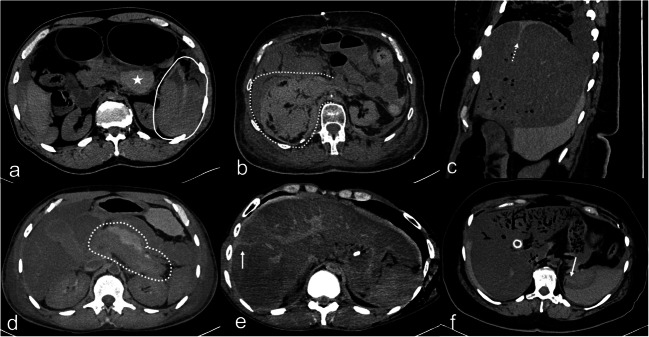
PMCT multiplanar reconstructions (MPR) in different cases with false positive (**a**–**d**) and one case with true positive (**e**) abnormal findings. **a** PMCT axial slice through the abdomen; false positive sedimented blood around the pancreas (asterisk) in a case of spontaneous splenic artery rupture (white line). **b** PMCT axial slice through the abdomen; false positive sentinel clot for the right kidney in a case of aortic rupture. **c** PMCT sagittal slice through the abdomen of the same case with f (details in f). **d** PMCT axial slice through the abdomen; false positive sentinel clot sign around the pancreas in a case where actually the liver and the spleen were injured. **e** PMCT axial slice through the abdomen of a case with true positive parenchymal disruption of the right liver lobe and sentinel clot sign around the liver. **f** PMCT-axial slice through the abdomen of a case with false positive parenchymal disruption of the liver (misinterpretation of a hepatic groove – dashed arrow in c). Note the true positive parenchymal disruption of the spleen (white arrow in f) with sedimented blood around the organ

**Fig. 4:**
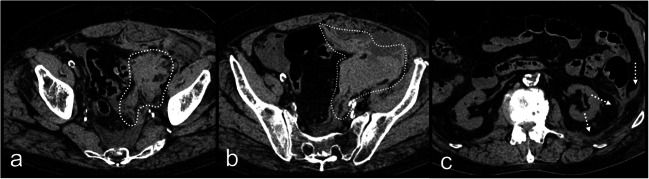
PMCT of a case with true positive findings for acute rupture of the left common femoral artery PMCT axial slices through the pelvis (**a**, **b**) and the retroperitoneal spaces at renal level (**c**); true positive sentinel clot sign of the left femoral artery. Note the accumulation of hyperdence fluid at the level of the artery in the pelvis (**a** and **b**), whereas the expanded fluid in the retroperitoneal spaces (**c**) is visually more hypodense

A total of 66.7% of the organs determined positive on PMCT based on the presence of the sentinel clot sign were TP (Table 3, Fig. 1, Fig. 2, Fig. 3, Fig. 4).

From the 44 FP in total, 13 (4 right kidneys, 1 pancreas, 7 spleens, and 1 left renal artery) were incorrectly determined positive based on the sentinel clot sign: sentinel clot was detected for the right kidney in one case of abdominal aortic rupture (Fig. 3), in one spleen laceration, in one liver laceration (but the fatty capsule of the right kidney exhibited hemorrhages at the autopsy), and in one case with liver and right adrenal gland injuries. FP sentinel clot was also determined for the pancreas in a case of spleen artery aneurysm rupture (Fig. 3). Interestingly, FP sentinel clot sign for the spleen was detected in 6 cases where actually the liver was the single organ injured. In another case, FP sentinel clot sign for spleen was determined where actually a splenic artery aneurysm rupture was the source. In a further case, sentinel clot sign was determined for the renal artery on PMCT; however, a spleen laceration was the actual bleeding site.

#### Blood or sedimented blood around the organ

**Fig. 5:**
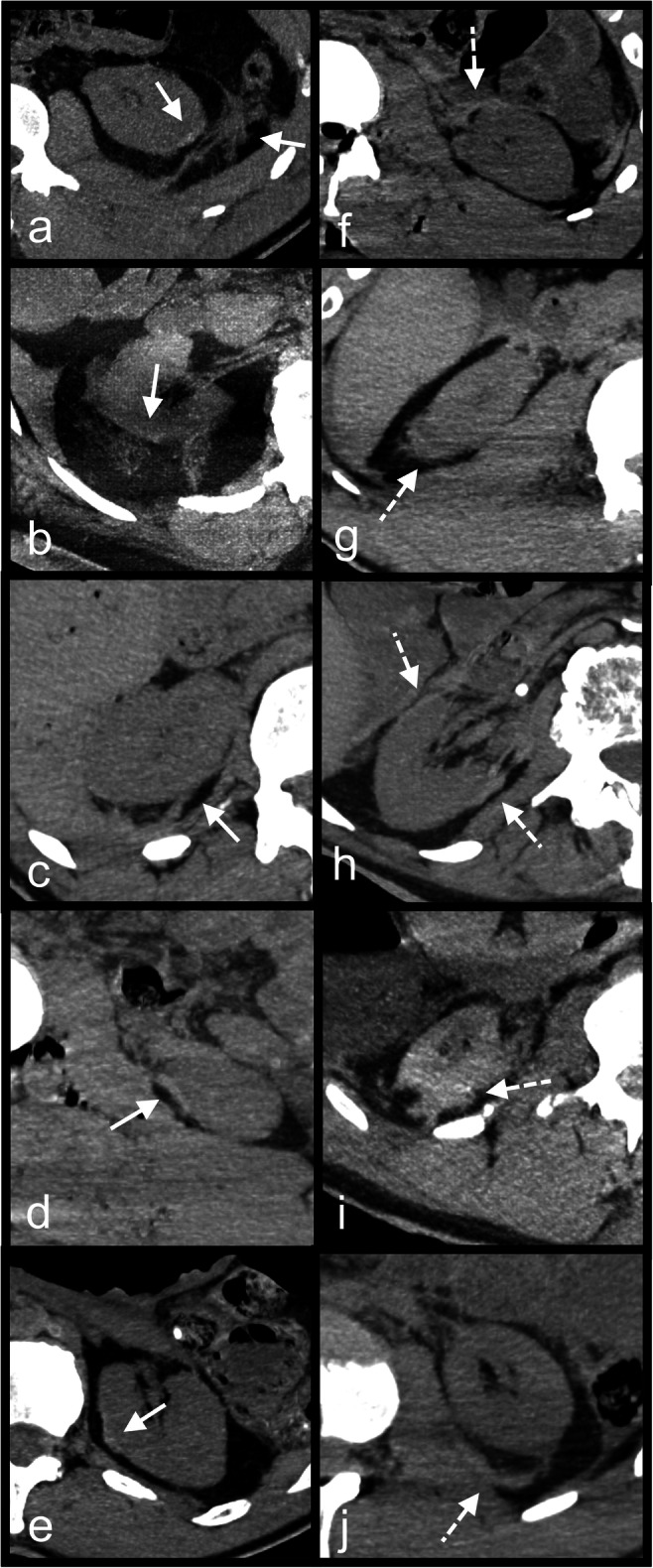
**a**–**e** PMCT axial slices through true positive kidneys (true positive findings with white arrows). **f**–**j** PMCT axial slices through false positive kidneys (false positive findings with white dashed arrows)

Twenty-one organs were FP based on the presence of surrounding blood and 6 based on the presence of surrounding sedimented blood (Fig. 1, Fig. 2). Two livers were considered as positive (one because of blood and one because of sedimented blood), whereas the spleen was actually the source in both cases (Fig. 3). Twelve spleens were FP where actually only the liver (in the 11 cases) or the liver and the right kidney (in one case) were bleeding. Interestingly, from the 4 cases with FP, sedimented blood surrounding the spleen, in 3 the liver, was the only source as result of resuscitation. Ten kidneys (6 right and 4 left) were FP based on presence of surrounding blood and blood in the retroperitoneal space, whereas only the liver (in 5 cases) or the liver and the spleen (in 5 cases) were actually bleeding bleeding (Fig. 5). In 3 cases, the pancreas was FP, whereas in the 2 of them, both the liver and spleen were injured and in one splenic aneurysm rupture was the source (Fig. 3).

#### Abnormal gas distribution and parenchymal disruption

A total of 91.3% of the organs with abnormal gas distribution on PMCT were TP (Fig. 1, Fig. 2). A total of 8.7% (*n* = 2) of them were FP, concerning 2 spleens with paradoxically suspected abnormal gas distribution on PMCT (confidence level for both spleens was equal to 3), whereas in another one, the liver and in another one the left kidney were injured. Regarding parenchymal disruption, only 2 organs showed parenchymal disruption as a single sign of possible injury on PMCT; the one (spleen) was TP but the other was FP (liver with confidence level = 3, where only the spleen was injured).

#### Combination of signs indicating bleeding source

Eleven organs showed a combination of signs on PMCT indicating bleeding source and all were TP (Fig. 1, Fig. 2, Fig. 4). Five showed the combination of sentinel clot with parenchymal disruption, 4 sentinel clot with abnormal gas distribution, 1 abnormal gas distribution with sedimented blood, and 1 abnormal gas distribution with parenchymal disruption.

### Raters’ confidence for detection of bleeding sources on PMCT

**Fig. 6:**
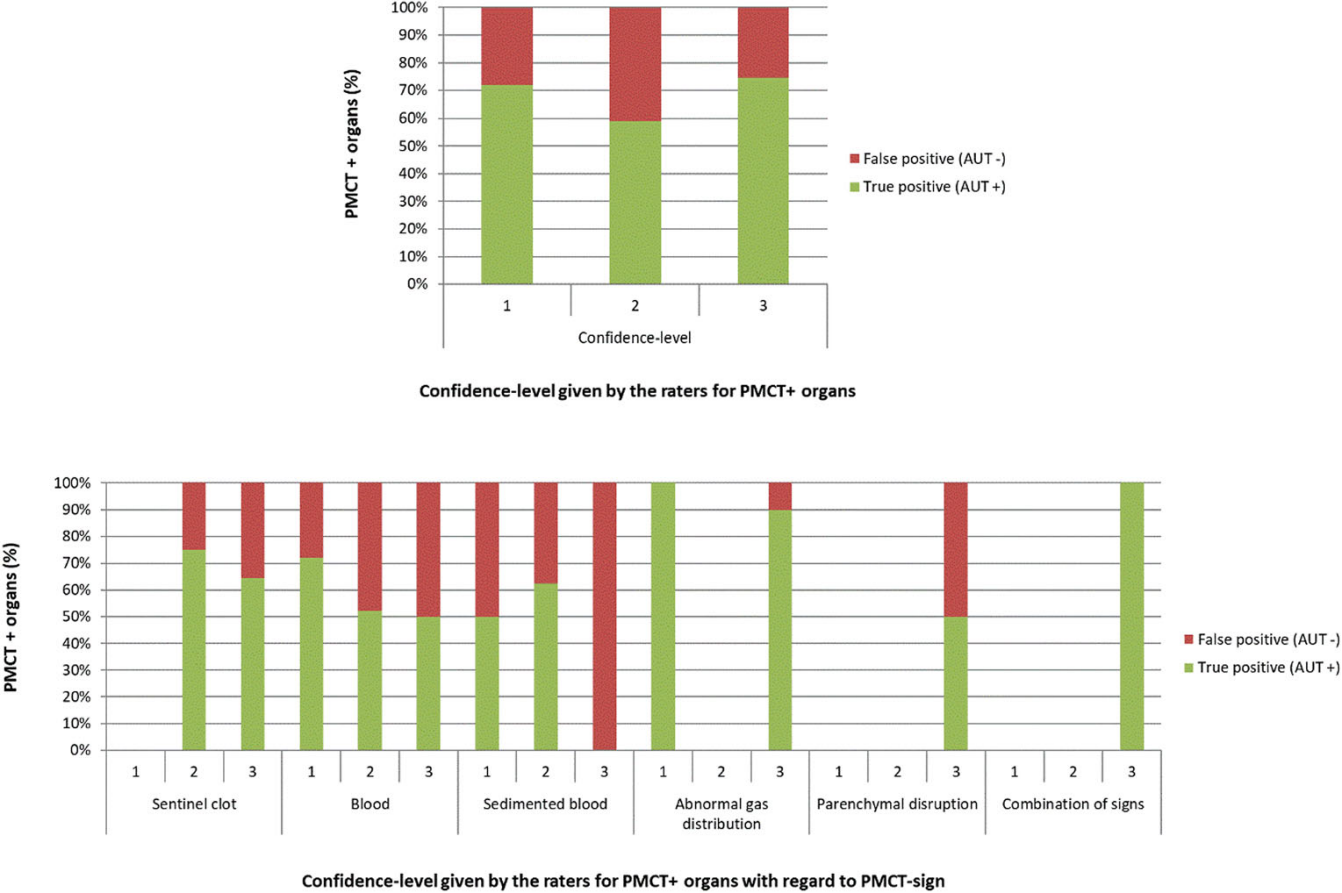
Bar charts depicting the frequencies (%) organs determined as positive on PMCT, i.e. the organs with at least one radiological sign on PMCT (“PMCT + organs”; true positive with green and false positive with red) with regard to the confidence level given by the raters (in consensus) when determined the organ as positive in total (chart on the top) and within the different PMCT signs (chart at the bottom). Higher confidence levels were not associated to higher proportions of true positive PMCT findings (chi-square test; *p* = 0.22)

Confidence levels given by the raters with regard to the five different radiological signs are presented in Fig. 6. Overall, independently of radiological sign, higher confidence levels were not associated to higher proportions of TP findings (chi-square test; *p* = 0.22).

### Radiological signs with regard to cause of intra-abdominal bleeding

**Fig. 7:**
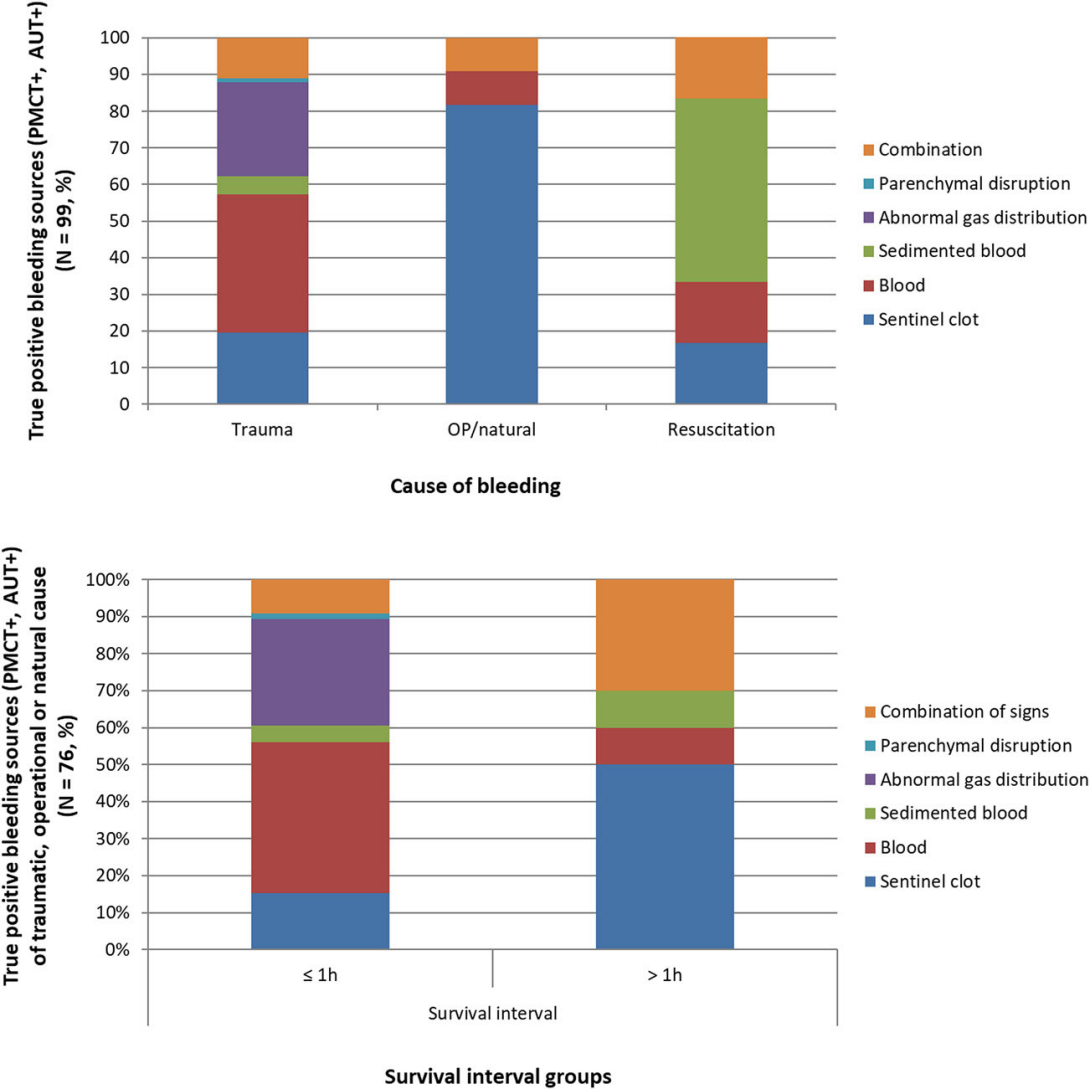
3 Graph on the top. Bar chart depicting the frequencies (%) of the PMCT signs of true positive organs (organs with at least one radiological sign on PMCT and positive at autopsy; “PMCT+, AUT+”, *n* = 99) with regard to the cause of bleeding (as determined at autopsy and based to case history). The distribution of the radiological signs was different among the cause of bleeding groups (Fischer’s exact test; *p* = 0.001). Graph at the bottom. Bar chart depicting the frequencies (%) of the PMCT signs of the true positive organs (organs with at least one radiological sign on PMCT and at autopsy: “PMCT+, AUT+”, *n* = 99) with cause of bleeding being trauma, operation or natural causes (and not resuscitation) (*n* = 76) in the two different survival interval groups (≤ 1 h or > 1 h). The distribution of the different PMCT signs was significantly different between the survival interval groups (Fischer’s exact test; *p* = 0.009)

From the 99 TP bleeding sources, 82 (82.8%) were result of trauma, 11 (11.1%) of a surgical or natural cause, and 6 (6.1%) of resuscitation measures. The distribution of the radiological signs was different among the cause of bleeding groups (Fischer’s exact test; *p* = 0.001, Fig. 7). TP bleeding sources that were caused by surgical or natural causes appeared most usually with the sentinel clot sign (81.8% within surgical/natural causes), whereas half (50%) of the TP bleeding sources caused by resuscitation appeared with surrounding sedimented blood. TP bleeding sources caused by trauma mainly appeared with blood in the region (37.8%), abnormal gas distribution (25.6%), sentinel clot (19.5%), and combination of signs (11%) (Fig. 7).

### Survival interval with regard to performance of PMCT and radiological signs

For the positive organs on PMCT, by which the bleeding was caused by trauma, surgery, or natural causes (and not resuscitation), the proportions of TP and FP findings were not different between the two survival interval groups (TP 75% in ≤ 1 h and 62.5% in > 1 h interval, FP 25% in ≤ 1 h and 37.5% in > 1 h interval, chi-square test; *p* = 0.36).

For the TP findings (*n* = 99), by which the bleeding was caused by trauma, surgical, or natural causes (*n* = 76), the distribution of the different radiological signs was significantly different between the survival interval groups (Fischer’s exact test; *p* = 0.009, Fig. 7). The sentinel clot sign appeared significantly more often in longer survival intervals (50% within > 1 h vs 15.2% within ≤ 1 h group). The same phenomenon was observed for sedimented blood, however with not so prominent differences. On the other hand, blood surrounding the bleeding source was a sign which appeared more often in shorter intervals (≤ 1 h 40.9% vs > 1 h 10%). Abnormal gas distribution and parenchymal disruptions were notably more in count in ≤ 1 h as single signs, as well as part in combination of signs, 6 cases with combination of signs in ≤ 1 h group, whereas 3 showed sentinel clot with parenchymal disruption, 2 sentinel clot with abnormal gas distribution, 1 parenchymal disruption with abnormal gas distribution, and 3 cases in >1 h group, whereas the 2 showed sentinel clot with parenchymal disruption and 1 abnormal gas distribution with sedimented blood around.

## Discussion

Apart from the overall diagnostic performance of PMCT, this study attempted to investigate the diagnostic performance of specific PMCT signs for the detection of the bleeding site in cases with hemoperitoneum. The presence of different signs was examined with regard to the cause of hemoperitoneum and survival interval. SEN was medium, while SPC was high for predicting the bleeding source. Different radiological signs are associated with different causes of hemoperitoneum and survival intervals.

The studied sample (85 cases with a total of 124 bleeding sites at autopsy) was relatively large compared to previous studies on PMCT [15, 16, 26]. The previous studies assessed PMCT accuracy for the detection of abdominal injury in road traffic accident victims [26] and traumatic deaths [15, 16]. Though the current study examined PMCT accuracy for the bleeding site only in cases with hemoperitoneum diagnosed at autopsy, the total SEN and SPC (79% and 92.1%, respectively) were similar to those found by Alvarez et al. (79.6% and 94.1%, respectively) [15]. However, the organ-specific SEN and SPC values of the current study were on average higher than those presented by Christe et al. (liver on PMCT alone, SEN 53%, SPC 84%; spleen on PMCT and magnetic resonance imaging (PMMR), SEN 50%, SPC 89%; kidneys on PMCT and PMMR, SEN 25%, SPC 100%) probably because of their smaller sample and because the reported organ injuries in their study were small and therefore difficult to detect in imaging [16]. SEN and SPC for liver and spleen were similar with that found by Norzailin et al. [26] and slight higher for the liver, spleen, and kidneys than those found by Juźwik et al. [27]. In a previous study with larger sample, injuries of pancreas were considered rare with PMCT providing medium sensitivity (71.4%) but high specificity (96.1%). In the current study, no pancreas injuries were detected at autopsy [27].

A total of 67% of the bleeding sources determined positive on PMCT based on the sentinel clot sign were TP. FP sentinel clot was very frequent for the right kidney and the spleen while other organs actually comprised the bleeding sites (most usually the liver). The sentinel clot sign was associated with natural and surgical causes, as well as with longer survival intervals (> 1 h). These observations may compound with the pathophysiological basis of the sign, which lies on the defending mechanism of the body against fatal blood loss outside the cardiovascular system. As hemostasis is attempted at the rupture site, a blood clot is formed, which provides a different radiological appearance than the lysed blood because of the different hemoglobin levels and subsequently different radiodensities [7]. It is assumed that clot formation requires a certain time and very short survival intervals may temporally not allow this hemostatic process to be sufficiently developed.

Blood and sedimented blood surrounding the assumed bleeding site were also TP for approximately 60% of the positive organs on PMCT. FP blood and sedimented blood accumulations around the spleen and the kidneys were usual in cases where actually only the liver or the liver and the spleen (in cases of FP kidneys) were the bleeding site. Blood accumulation was associated with short survival intervals (≤ 1 h), whereas sedimented blood with larger ones (> 1 h). This can be explained by the fact that the cellular blood components are allowed to sink and sediment within the vascular system and cavities (i.e., hemothoraces, hemoperitoneum) over time. This phenomenon may be not fully developed in very short survival intervals. Early sedimentation may be associated with longer agonal intervals (“In individuals dying a slow lingering death with terminal cardiac failure, livor mortis may actually appear antemortem” [24]). Interestingly, sedimented blood around the bleeding site was more usual in cases where bleeding was caused by resuscitation. This observation conforms to observations about hemopericardium’s radiological appearance in cases with antemortem (associated with double-band sign) and cases with hemopericardium caused by postmortem manipulations and resuscitation (single-band sign or horizontal level) [28, 29].

It seems that in cases where clot is not clearly or not at all formed and subsequently invisible on PMCT, the blood floats and may be re-distributed in the abdominal cavity (i.e., from the right subphrenic to the left subphrenic space and vice versa or from the subphrenic spaces through the paracolic gutters into the pelvis). There exists communication between the retrohepatic space and the retroperitoneal perirenal space (so-called Kneeland channel) [30], which also allows the transportation of blood from the peritoneal to the retroperitoneal spaces, and this can explain the large number of FP kidney signs where actually only the liver or the liver and the spleen or the abdominal aorta was bleeding.

Abnormal intraparenchymal gas distribution not following the vascular tree was TP for 90% of the positive cases on PMCT and was associated with traumatic causes and short survival intervals (≤ 1 h). It is assumed that injuries appearing with abnormal gas distribution are related to more severe and life-threatening conditions and can subsequently be more easily visible on PMCT, increasing SEN [16]. The same was observed for parenchymal disruptions. It was reasonable to expect that combinations of radiological signs would increase the PMCT sensitivity for the detection of the bleeding source and indeed all organs determined as possible bleeding sites based on a combination of signs on PMCT were TP.

This study has limitations. Not all intraperitoneal organs were evaluated as possible bleeding sources. Parts of the gastrointestinal tract, the internal genital organs, gall, and urinary bladder were not assessed under the assumption that after their perforation, the consistency of the intraperitoneal fluid is not pure blood. Secondly, no pancreatic injuries were detected in this sample; thus, accuracy values for pancreas could not be extracted. Thirdly, although the raters were blinded to autopsy findings, detection bias may exist as they were aware that hemoperitoneum was present on all reviewed PMCT. However, the aim of the study was to determine the PMCT accuracy for the detection of the bleeding source and not for the detection of hemoperitoneum itself. Last but not the least, because of the retrospective manner of this study, a reliable extraction of information regarding possible medical handlings (infusions, transfusions, etc.) between incident and time of death was not possible based on the review of the autopsy reports, which may have brought bias in the estimation of the presented survival intervals and maybe also in the postmortem radiological appearance of hemoperitoneum. However, this condition may correspond to real conditions considering that such information often lacks in the forensic routine at the time a forensic radiologist is called to determine a possible bleeding source on PMCT.

## Conclusion

PMCT provides moderate sensitivity and high specificity for the detection of abdominal bleeding sources in postmortem cases with hemoperitoneum. For the true positive organ findings, specific radiological signs may be associated to the different causes of bleeding and the survival interval.
